# Life-Threatening Metformin-Induced Lactic Acidosis Associated With Euglycemic Ketoacidosis and Acute Multiorgan Dysfunction: A Case Report

**DOI:** 10.7759/cureus.82717

**Published:** 2025-04-21

**Authors:** Karim S Hussein, Rashid Nadeem, Ahmad Nabil

**Affiliations:** 1 Critical Care Medicine, Dubai Hospital - Dubai Health, Dubai, ARE; 2 Intensive Care Medicine, Dubai Hospital - Dubai Health, Dubai, ARE; 3 Anesthesia, Dubai Hospital - Dubai Health, Dubai, ARE; 4 Anesthesia and Intensive Care, Ain Shams University, Cairo, EGY

**Keywords:** acute renal injury, cardiac arrest outcome, continuous venovenous hemodialysis (cvvhd), diabetic ketoacidosis (dka), metformin associated gastrointestinal intolerance, metformin induced lactic acidosis, refractory lactic acidosis, refractory metabolic acidosis

## Abstract

Metformin-associated lactic acidosis (MALA) is a rare but life-threatening complication of metformin therapy. We present a case of a 63-year-old female with type 2 diabetes mellitus (on metformin and insulin) who developed severe lactic acidosis, euglycemic diabetic ketoacidosis (DKA), and acute kidney injury (AKI) following a three-day history of gastrointestinal symptoms. Despite initial stabilization efforts, the patient deteriorated into refractory shock and cardiac arrest, requiring intensive care unit (ICU) admission, continuous venovenous hemodiafiltration (CVVHD), vasopressor support, and mechanical ventilation. Serial arterial blood gas (ABG) analyses demonstrated profound metabolic acidosis (pH 6.77, lactate 20 mmol/L) with gradual normalization following CVVHD. The patient recovered fully, highlighting the importance of early recognition and aggressive management of MALA, including renal replacement therapy (RRT), in critically ill patients.

## Introduction

Metformin, a cornerstone therapy for type 2 diabetes mellitus, is widely regarded as safe, yet it carries a rare but life-threatening risk of lactic acidosis. Studies like the UK Diabetes Study [1] and insights from Peters et al. [2] highlight the contentious nature of managing metformin-associated lactic acidosis (MALA). Treatment options range from supportive care and activated charcoal to bicarbonate infusions, hemodialysis, or continuous venovenous hemofiltration. Peters and colleagues previously reported in Critical Care that mortality rates in MALA patients admitted to the ICU were comparable between those who received dialysis and those who did not. However, dialysis was often reserved for the sickest patients - those with acute and chronic comorbidities - suggesting it may have mitigated an otherwise higher mortality risk. In rare instances, metformin toxicity can spiral into multiorgan failure, driven by renal impairment and lactic acidosis, as illustrated in this case.

Our aim

To present a rare and life-threatening case of MALA complicated by euglycemic diabetic ketoacidosis (DKA) and acute multiorgan dysfunction, highlighting the diagnostic and therapeutic challenges in its management.

## Case presentation

A 63-year-old woman with a history of hypothyroidism (managed with levothyroxine), hypertension (controlled with medication), morbid obesity, and type 2 diabetes (treated with insulin and metformin) arrived at the emergency department (ED) after three days of fatigue, nausea, vomiting, diarrhea, and profound weakness. Paramedics found her hypoglycemic (glucose 17 mg/dL) and administered glucose en route. She reported severe abdominal pain, distention, and polyuria over the prior two days but denied fever, chest pain, respiratory issues, or headache. Notably, she had skipped her medications on the day of admission.

Presentation

Upon arrival in the ED, the patient appeared lethargic yet remained fully alert, with a Glasgow Coma Scale score of 15/15, and showed clear signs of severe dehydration. Despite normalization of her blood glucose levels, she was hypotensive and exhibited alarming metabolic abnormalities, including elevated ketones, severe metabolic and lactic acidosis, acute kidney injury (AKI), and anuria. Thyroid function tests were normal, while septic markers showed only mild elevation.

Management and progression

Resuscitation began immediately with intravenous fluids, vasopressors, sodium bicarbonate, and a DKA protocol. Despite these aggressive measures, her condition deteriorated into refractory shock, requiring dual vasopressor support. She experienced a cardiac arrest in the ED but was successfully revived after two cycles of CPR and intubation. Imaging studies, including CT of the brain, abdomen, and pelvis, as well as a chest X-ray, were performed to identify potential causes of her decline. Suspecting metformin toxicity due to persistent lactate levels above 20 mmol/L and severe acidosis - despite negative cultures - she was transferred to the ICU for urgent continuous venovenous hemodiafiltration (CVVHD).

**Figure 1:**
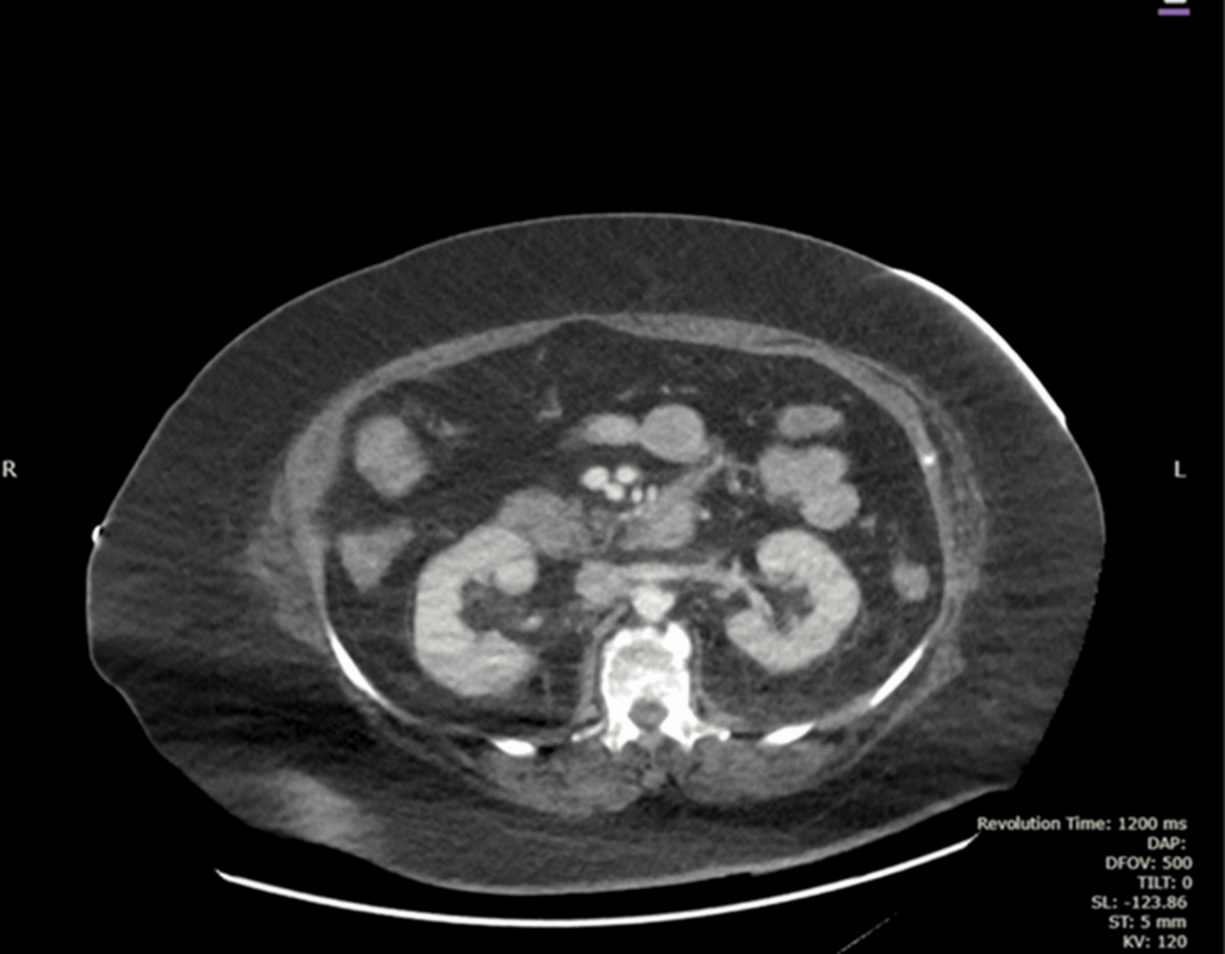
CT Abdomen with Contrast Pancreas is normal in size with normal shape

**Figure 2:**
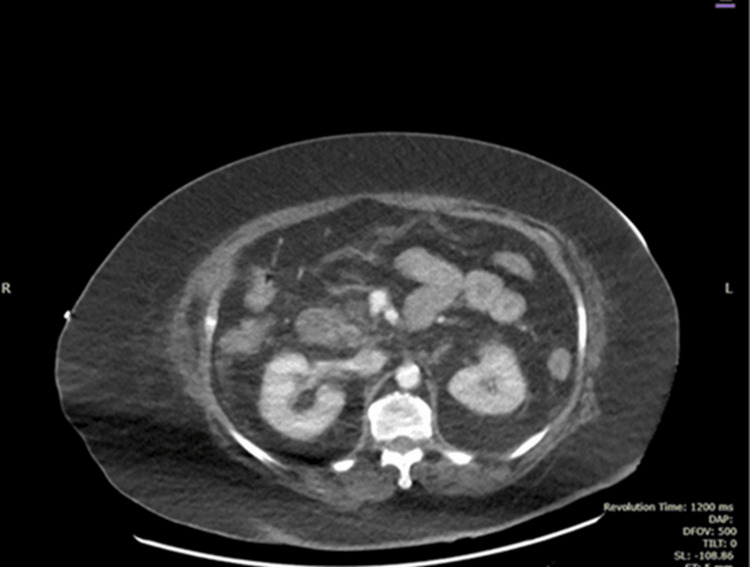
CT Abdomen with Contrast No active intestinal ischemia

**Figure 3:**
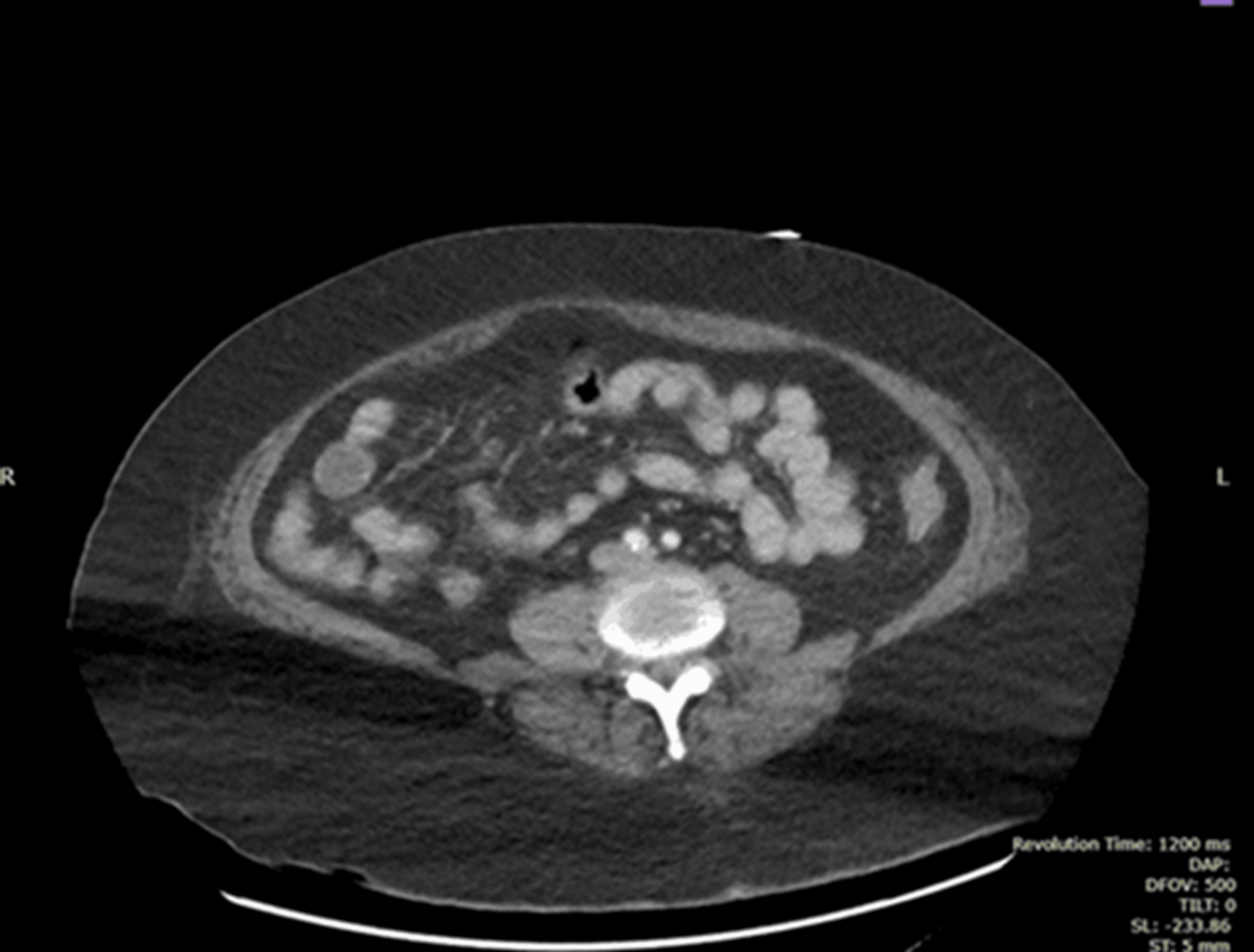
CT Abdomen with Contrast No intestinal ischemia

Imaging revealed several findings. The CT of the abdomen and pelvis with contrast showed a normal pancreas in size and shape, with no evidence of focal lesions or ductal abnormalities (Figure 1). Additionally, no active intestinal ischemia was observed (Figures 2, 3). The kidneys appeared normal in size and enhancement, without hydronephrosis or lesions, while diffuse colonic wall thickening with uncomplicated diverticula suggested an infectious process. The left adrenal gland was bulky, though the right adrenal was unremarkable, and no pneumoperitoneum, ascites, or intra-abdominal adenopathy was observed. Atheromatous calcification was noted in the aorta and its branches, and left basal consolidation and cardiomegaly were also evident.

**Figure 4:**
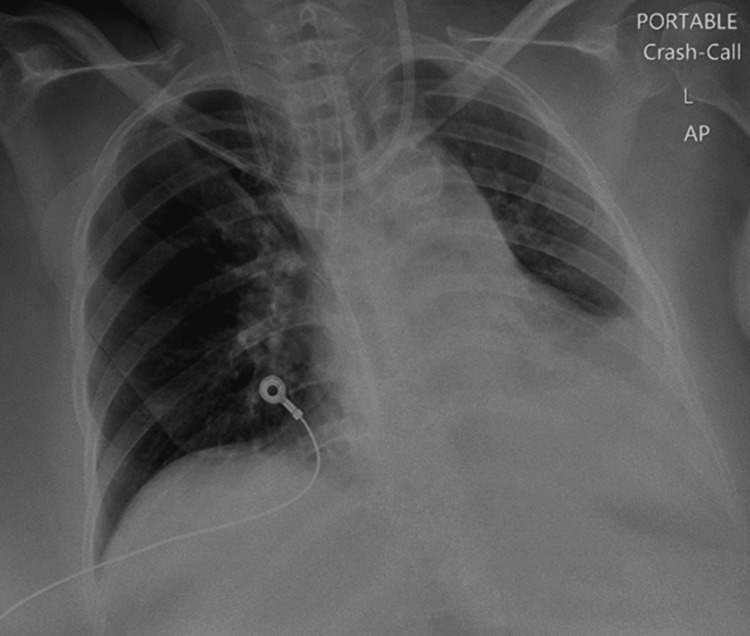
Chest X-Ray The chest X-ray revealed that the endotracheal tube was positioned low in the right main bronchus, necessitating adjustment, while the left costophrenic angle was obscured by the cardiac shadow. No airspace opacities or pneumothorax were identified.

The chest X-ray revealed that the endotracheal tube was positioned low in the right main bronchus, necessitating adjustment, while the left costophrenic angle was obscured by the cardiac shadow. No airspace opacities or pneumothorax were identified (Figure 4).

In the ICU, CVVHD was initiated alongside ventilatory support and triple vasopressor therapy to stabilize her hemodynamics. Within 24 hours, her ketones normalized, urine output exceeded 1 mL/kg/hour, and lactate levels began to decline, reaching normal by day three. Serial arterial blood gas (ABG) and lactate measurements tracked her progress, as shown in Table 1.

**Table 1:** Serial Arterial Blood Gas (ABG) With Lactate Levels From Admission to Pre-extubation pO2: partial pressure of oxygen; pCO2: partial pressure of carbon dioxide; HCO3: Bicarbonate

Parameter	Reference Range	On Admission	12 Hours	24 Hours	36 Hours	48 Hours	72 Hours
pH	7.35–7.45	6.77	7.05	7.19	7.25	7.38	7.46
pO2 (kPa)	10–13.3 (75–100 mmHg)	16.7	15.4	14.2	12.4	9.2	10
pCO2 (kPa)	4.7–6.0 (35–45 mmHg)	2.17	2.19	2.8	3.1	3.6	3.9
HCO3 (mmol/L)	22–26	5	5.2	8.4	14.0	18.8	28.6
Base Excess (BE)	-2 to +2	-27.9	-27.7	-24.5	-21.2	-4.2	-2.3
Lactate (mmol/L)	0.5–2.0	20	12.5	9	6.1	3.2	2.6

Outcome

By day four in the ICU, the patient was weaned off vasopressors and ventilatory support, becoming fully awake and communicative. Her renal function improved steadily, eliminating the need for further CVVHD. After one additional day of observation in the ICU, she was transferred to the medical ward under nephrology care and was discharged home after 13 days in the hospital.

Notable observations

During her hospital stay, her hemoglobin dropped from 13 g/dL to 9.5 g/dL over seven days without evidence of bleeding, but it spontaneously recovered to 12 g/dL within weeks. Additionally, mildly elevated amylase and lipase levels were noted on admission, though no clear cause was identified. This case underscores the urgency of recognizing and treating euglycemic DKA and metformin toxicity in vulnerable patients with severe metabolic compromise.

## Discussion

MALA is a rare but potentially lethal complication of a drug otherwise celebrated for its efficacy and safety in type 2 diabetes management. The diagnostic criteria for MALA hinge on a history of metformin exposure coupled with lactic acidosis, defined here as a lactate concentration ≥5 mmol/L and bicarbonate <22 mmol/L at or before ICU admission. While plasma metformin levels can confirm toxicity, their routine measurement is neither widely available nor clinically essential, as metformin exerts its toxic effects intracellularly. Even at therapeutic concentrations (0.5-1 mg/L fasting, 1-2 mg/L postprandial), metformin can disrupt lactate metabolism by inhibiting pyruvate carboxylase, reducing glucose utilization, and increasing hepatocyte lactate production [3]. This case exemplifies how such metabolic derangements, compounded by acute stressors like gastroenteritis and dehydration, can precipitate a cascade of renal failure, acidosis, and multiorgan dysfunction.

The patient’s presentation with euglycemic DKA - a state of ketosis without hyperglycemia - further complicates the diagnostic landscape. Euglycemic DKA is an underrecognized entity, often linked to factors like reduced carbohydrate intake, vomiting, or, as in this case, metformin’s interference with glucose metabolism. Her severe gastroenteritis likely exacerbated metformin accumulation by causing hypovolemia and AKI, impairing the drug’s primary route of elimination - renal excretion. This synergy of factors underscores the importance of considering MALA in any metformin-treated patient presenting with unexplained acidosis, even in the absence of overt hyperglycemia.

Therapeutic approaches to MALA remain debated, as highlighted by Finkle [1]. Options include gastrointestinal decontamination (e.g., activated charcoal), sodium bicarbonate to correct acidosis, and renal replacement therapies like hemodialysis or CVVHD. In this case, CVVHD proved pivotal, rapidly clearing lactate and stabilizing the patient’s hemodynamics despite initial refractory shock and cardiac arrest. The cardiac toxicity of uncorrected acidemia, as noted by Rodríguez-Villar et al., arises from impaired myocardial contractility, a risk that timely correction of acid-base balance can potentially mitigate [4]. Notably, the patient’s negative cultures ruled out sepsis as a primary driver, reinforcing metformin toxicity as the central etiology.

The broader literature offers conflicting insights into MALA’s prognosis. Peters et al. observed similar mortality rates between dialyzed and non-dialyzed patients, yet dialysis was disproportionately used in sicker individuals, suggesting a survival benefit in severe cases [2]. In our patient, the combination of CVVHD, ventilatory support, and vasopressors reversed a dire trajectory, with lactate normalizing within 72 hours and renal function recovering without further intervention. This aligns with reports that early, aggressive management can avert fatal outcomes, particularly when multiorgan failure looms.

Additional observations, such as the transient hemoglobin drop (13 g/dL to 9.5 g/dL) without bleeding, warrant consideration. While metformin-induced hemolytic anemia has been documented [3,5], the spontaneous recovery here suggests hemodilution or stress-related marrow suppression rather than a direct drug effect. In contrast, diagnostic tools like the direct antiglobulin (Coombs’) test, used to detect immune-mediated hemolysis in newborns [6], are less relevant in this adult context, where no evidence of hemolysis (e.g., schistocytes, elevated bilirubin) emerged. Similarly, the mild elevation in amylase and lipase, without imaging evidence of pancreatitis, may reflect a nonspecific stress response or subtle metformin-related gastrointestinal injury [7]. These findings highlight the multisystem impact of MALA and the need for comprehensive monitoring beyond acid-base status.

This case also raises questions about risk stratification and prevention. Metformin’s excellent enteral bioavailability and renal clearance make it vulnerable to accumulation in settings of dehydration, renal impairment, or overdose. Clinicians must remain vigilant for prodromal symptoms like gastroenteritis, which can tip a stable patient into crisis. Patient education on medication adherence and prompt reporting of such symptoms could further reduce MALA’s incidence, though its rarity complicates broad screening efforts.

## Conclusions

A 63-year-old woman with type 2 diabetes developed severe MALA with euglycemic diabetic ketoacidosis and multiorgan dysfunction, precipitated by gastroenteritis. Presenting with profound acidosis (pH 6.77, lactate 20 mmol/L) and shock, she required urgent CVVHD, vasopressors, and ventilation post-cardiac arrest. Full recovery highlights the necessity of early MALA recognition in metformin users with gastrointestinal symptoms and prompt CVVHD for severe cases, alongside patient education to prevent recurrence.
